# A high-mobility electronic system at an electrolyte-gated oxide surface

**DOI:** 10.1038/ncomms7437

**Published:** 2015-03-12

**Authors:** Patrick Gallagher, Menyoung Lee, Trevor A. Petach, Sam W. Stanwyck, James R. Williams, Kenji Watanabe, Takashi Taniguchi, David Goldhaber-Gordon

**Affiliations:** 1 Department of Physics, Stanford University, Stanford, California 94305, USA; 2 Department of Applied Physics, Stanford University, Stanford, California 94305, USA; 3 Advanced Materials Laboratory, National Institute for Materials Science, 1-1 Namiki, Tsukuba 305-0044, Japan

## Abstract

Electrolyte gating is a powerful technique for accumulating large carrier densities at a surface. Yet this approach suffers from significant sources of disorder: electrochemical reactions can damage or alter the sample, and the ions of the electrolyte and various dissolved contaminants sit Angstroms from the electron system. Accordingly, electrolyte gating is well suited to studies of superconductivity and other phenomena robust to disorder, but of limited use when reactions or disorder must be avoided. Here we demonstrate that these limitations can be overcome by protecting the sample with a chemically inert, atomically smooth sheet of hexagonal boron nitride. We illustrate our technique with electrolyte-gated strontium titanate, whose mobility when protected with boron nitride improves more than 10-fold while achieving carrier densities nearing 10^14^ cm^−2^. Our technique is portable to other materials, and should enable future studies where high carrier density modulation is required but electrochemical reactions and surface disorder must be minimized.

## Abstract (web summary)

Electrolyte gating enables the accumulation of large carrier densities in two-dimensional electron systems. Here, the authors demonstrate that a few-atom thick layer of hexagonal boron nitride can dramatically improve carrier mobility in an electrolyte-gated system by limiting chemical reactions and disorder.

## Graphical abstract

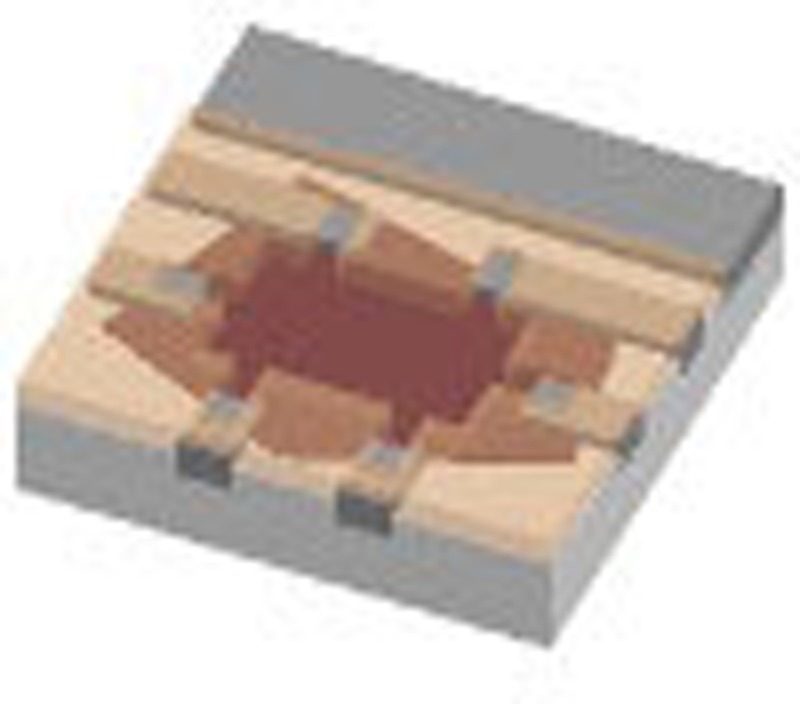

A conventional field effect transistor is controlled by the voltage on a metal electrode separated from the channel by a thin insulating dielectric. The maximum applied voltage is determined by the dielectric breakdown field, beyond which the resistance of the dielectric sharply drops, shorting the metal electrode to the channel. For a typical high-quality dielectric, the breakdown field limits the accumulated carrier density to ~10^13^ cm^−2^ (ref. 1), although for special cases such as ferroelectrics stronger modulation is possible^2^,^3^,^4^. Electrolyte gating circumvents dielectric breakdown by eliminating the metal/dielectric interface: an electrolyte is applied directly to the surface of interest and polarized, drawing one charged species to the surface and building a large electric field^5^. Carrier densities ~10^15^ cm^−2^ can be induced by electrolyte gating^6^, facilitating the discovery of superconductivity in new parameter regimes^7^,^8^ and the creation of novel photonic devices^9^, among other advances.

While very effective at modulating surface properties, electrolyte gating also introduces disorder. The deposition of contaminants on the sample is difficult to control, a problem that is compounded by the possibility of surface-degrading electrochemical reactions. Recent studies have further suggested that chemical modification of the surface of interest, rather than electrostatics, is primarily responsible for the marked changes in electronic properties in some electrolyte-gated systems^10^,^11^,^12^.

Motivated by these challenges, we consider the well-studied two-dimensional electron system (2DES) created by electrolyte gating at the surface of strontium titanate (STO)^13^,^14^,^15^,^16^,^17^,^18^,^19^. The transport properties of this surface 2DES closely resemble those of the 2DES at the lanthanum aluminate/strontium titanate (LAO/STO) interface. However, the highest reported low-temperature electron mobility in the STO 2DES is about 1,000 cm^2^ V^−1^ s^−1^, at an electron density of 3 × 10^13^ cm^−2^ (refs 13, 14, 16, 19); for the same density, the LAO/STO 2DES has mobility up to 10,000 cm^2^ V^−1^ s^−1^ (ref. 20). We demonstrate that by protecting the STO channel with a thin boron nitride (BN) dielectric impermeable to the ions of the electrolyte^21^, the mobility of the resulting electrolyte-gated 2DES substantially increases over a wide density range, surpassing 12,000 cm^2^ V^−1^ s^−1^ at a density of 4 × 10^13^ cm^−2^ in our best sample.

## Results

### BN-protected STO samples

**Figure 1:**
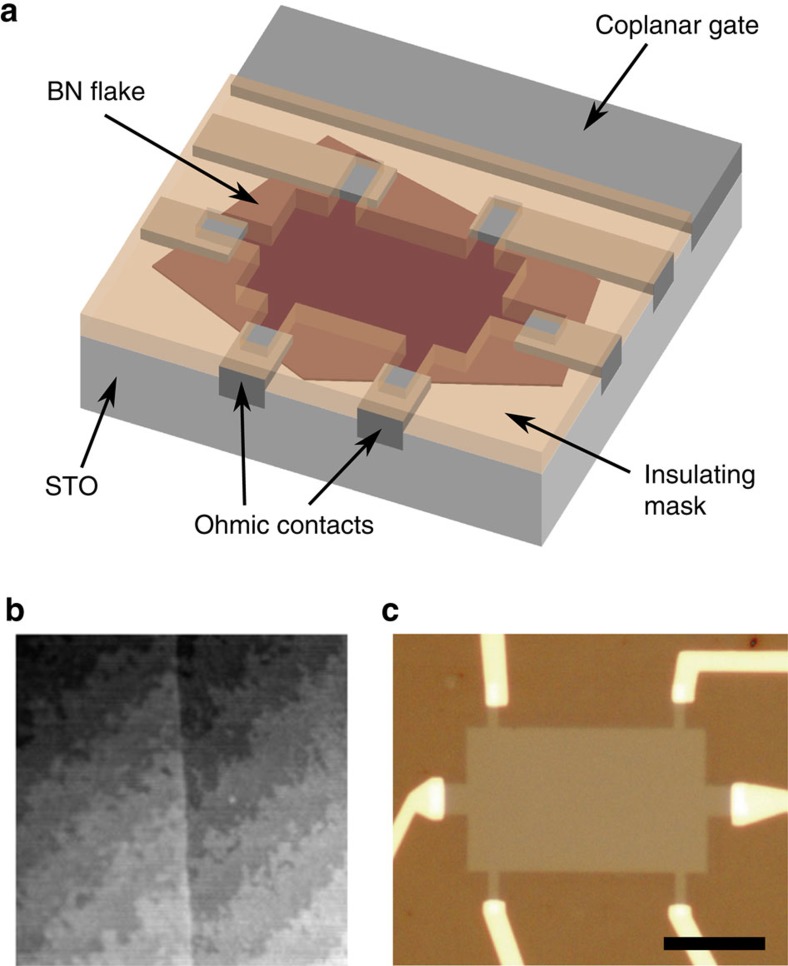
Electrolyte gating with a boron nitride barrier. (**a**) Schematic representation of a device fabricated on a single crystal of strontium titanate (STO). In operation, the entire device is submerged in ionic liquid (not shown), which is polarized by the coplanar gate. (**b**) Atomic force micrograph (topography) of a few-layer boron nitride (BN) flake (left half of image) on an STO crystal. STO terraces (0.4-nm steps) run bottom left to top right, and are visible beneath the BN, indicating that the flake conforms to the substrate with few trapped impurities. Scan window is 1 μm by 1 μm. (**c**) Optical micrograph of Sample A, which has a cross-linked PMMA mask (darker brown regions; the relative lightness here is opposite to that in **a**, where to aid visualization the flake is darker than the mask). The thin BN flake is not visible on STO, but covers the entire opening in the PMMA mask, except near the contacts. Scale bar: 10 μm.

Each of our samples consists of a single crystal of STO partially covered by an atomically flat BN flake (Fig. 1a). The BN flake conforms to the substrate without trapping contaminants, as evidenced by the 0.4 nm terrace steps of the underlying STO seen in the topography of the BN (Fig. 1b). The substrate is masked by a thick insulator except in a Hall bar-shaped channel area (Fig. 1c); the electrolyte induces negligible carrier density in the masked regions. In this work, we consider four BN-covered STO samples—denoted A, B, C and D—with BN thicknesses measured to be 0.6, 1.0, 1.2 and 1.5 nm, respectively, by atomic force microscopy (see Supplementary Note 1 for lateral dimensions and thickness measurement details). For each sample, we collect low-temperature magnetotransport data over multiple cooldowns at different coplanar gate voltages *V*_gate_.

### Mobility and carrier density

**Figure 2:**
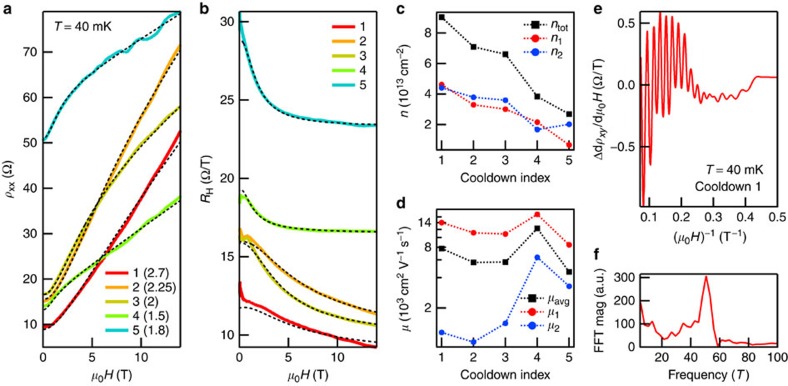
High-mobility magnetotransport in Sample A over a range of densities. (**a**) Sheet resistance *ρ*_xx_ (symmetrized in field) for 5 cooldowns, labelled 1, 2, 3, 4 and 5, with *V*_gate_=2.7, 2.25, 2, 1.5 and 1.8 V, respectively. Dashed black curves result from two-band fits with linear magnetoresistance, performed simultaneously on the data in **a** and **b**. The BN is 0.6 nm thick. (**b**) Hall coefficient *R*_H_≡*ρ*_xy_/*μ*_0_*H* (symmetrized in field) for the same 5 cooldowns as in **a** and fits (dashed black curves). (**c**) Extracted carrier densities *n*_1_ and *n*_2_ for the two bands for each cooldown; *n*_tot_=*n*_1_+*n*_2_. (**d**) Extracted carrier mobilities *μ*_1_ and *μ*_2_ for the two bands for each cooldown; *μ*_avg_=(*n*_1_*μ*_1_+*n*_2_*μ*_2_)/*n*_tot_. (**e**) Quantum oscillations in *dρ*_*xy*_/*dμ*_0_*H* as a function of inverse applied magnetic field for Cooldown 1. For clarity, the signal has been smoothed and a quadratic background has been subtracted. The oscillations commence at ~3 T for all cooldowns. (**f**) Magnitude of the Fourier transform of **e**, showing a peak at ~50 T, corresponding to a density ~2 × 10^12^ cm^−2^.

The striking improvement in 2DES quality with a BN spacer is evident in the magnetotransport properties of Sample A, which is covered by a 0.6-nm thick flake (Fig. 2a,b). The five cooldowns of Sample A, numbered 1 through 5, correspond to different *V*_gate_ settings. Although higher *V*_gate_ typically induces higher density, this is not always the case because of hysteresis (see Methods) and because of drifting offset voltages from electrochemical reactions at the gate electrode. To extract density and mobility, we perform a simultaneous fit to the sheet resistance *ρ*_xx_ and the Hall coefficient *R*_H_≡*ρ*_xy_/*μ*_0_*H*, where *ρ*_xy_ is the Hall resistance, *μ*_0_ is the magnetic constant, and *H* is the applied magnetic field. As is typical in the STO 2DES literature, we assume that the magnetotransport behaviour can be described by two bands^22^. Although quantum oscillation data suggest several bands (discussed below), a two-band description often fits the data, providing reliable numbers for average mobility and total density (Supplementary Note 2). For LAO/STO, a two-band fit with four parameters (densities *n*_1_, *n*_2_, mobilities *μ*_1_, *μ*_2_) captures the approximate shapes of *ρ*_xx_ and *R*_H_, but deviates from the data at higher fields^22^. We encounter the same difficulty: the two-band model cannot simultaneously fit the nonsaturating linear magnetoresistance and nearly saturated Hall coefficient observed up to 31 T in our samples (Supplementary Note 2) and in LAO/STO samples^23^. Inclusion of a third band cannot generally reproduce our high-field data, and where a three-band fit does work, the required densities are unrealistically large, frequently exceeding 10^16^ cm^−2^ with mobility ~1 cm^2^ V^−1^ s^−1^. We instead fit to a two-band model in which the sheet resistance of each band contains a term linear in applied field: *ρ*_xx,i_=1/*n*_*i*_*eμ*_*i*_+*k*_*i*_*H* for *i*=1, 2 and *k*_*i*_≥0. The linear term could arise from spatial fluctuations in mobility^24^,^25^.

Our two-band fits with linear magnetoresistance provide an excellent match to the data (Fig. 2a,b). These fits exclude the low-field region, where the magnetotransport properties are affected by magnetic moments in the STO (ref. 26 and Supplementary Note 2). We find a high-mobility band with density *n*_1_ between 6 × 10^12^ and 5 × 10^13^ cm^−2^ (Fig. 2c) and mobility *μ*_1_ between 8,000 and 17,000 cm^2^ V^−1^ s^−1^ (Fig. 2d), as well as a low-mobility band with a similar density *n*_2_ and mobility *μ*_2_ that grows with decreasing *n*_2_. The total induced density *n*_tot_ can reach 9 × 10^13^ cm^−2^ (Fig. 2c) with an average mobility *μ*_avg_=(*n*_1_*μ*_1_+*n*_2_*μ*_2_)/*n*_tot_ approaching 8,000 cm^2^ V^−1^ s^−1^ (Fig. 2d). The average mobility for Cooldown 4 exceeds 12,000 cm^2^ V^−1^ s^−1^. These mobilities match (for lower densities) and exceed (for higher densities) the highest reported mobilities in LAO/STO 2DES^20^,^27^, and are 10 times larger than the mobilities reported in the literature for electrolyte-gated STO 2DES at any carrier density^13^,^14^,^19^. Our conclusions are unchanged if we instead calculate *μ* and *n* by naively dividing *R*_H_ by *ρ*_xx_, or if we fit with the four-parameter, two-band model (Supplementary Note 2).

### Quantum oscillations

Quantum oscillations appear above ~3 T in both *ρ*_xx_ and *ρ*_xy_ for all cooldowns. The *ρ*_xy_ oscillations from Cooldown 1 (Fig. 2e) show a primary oscillation frequency of 50 T (Fig. 2f), corresponding to a carrier density near 2 × 10^12^ cm^−2^. This contrasts with the results of the two-band Hall transport fits, in which both bands are at least 10 times more populated. For a typical cooldown, we can identify multiple quantum oscillation frequencies corresponding to densities ~10^12^ cm^−2^, regardless of the total density measured by the Hall effect. The strongest oscillations thus appear for the lowest Hall densities (see Cooldown 5 in Fig. 2a), as the bands that produce quantum oscillations now constitute a substantial fraction of the carriers. Our findings resemble quantum oscillation data collected on the highest mobility LAO/STO 2DES, in which multiple bands of density ~10^12^ cm^−2^ show quantum oscillations, and total Hall densities ~10^13^ cm^−2^ or lower are required for strong oscillations in *ρ*_xx_ (refs 28, 29). The presence of low-density oscillating bands does not strongly impact the shapes of *ρ*_xx_ and *R*_H_, so the two-band model still captures most of the device behaviour (Supplementary Note 2).

## Discussion

**Figure 3:**
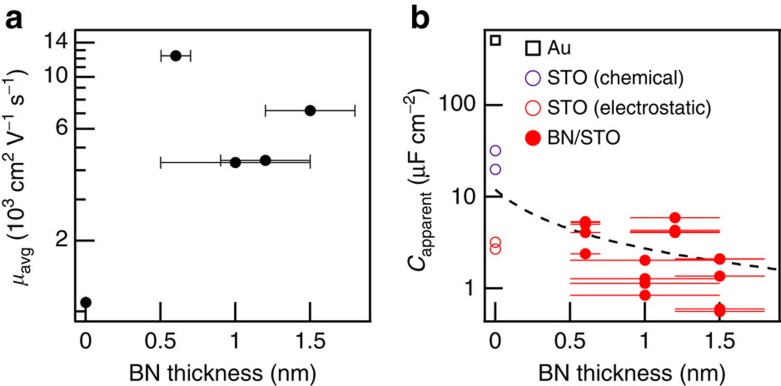
Properties of all measured samples. (**a**) Best average mobility *μ*_avg_, extracted from two-band fits with linear magnetoresistance, recorded over all cooldowns at the various boron nitride (BN) thicknesses studied. We have included our highest mobility uncovered strontium titanate (STO) sample (BN thickness zero). Error bars indicate thickness uncertainty in our atomic force microscope measurements (Supplementary Note 1). (**b**) Apparent capacitance *C*_apparent_=*en*_tot_/*V*_gate_ versus BN thickness for all cooldowns on all BN-covered STO samples (filled red circles). The total density *n*_tot_ is extracted from two-band fits with linear magnetoresistance. Dashed black line is the maximum capacitance *C*_max_ for electrostatic carrier accumulation. For comparison, we include *C*_apparent_ for the bare STO samples from ref. 14; those which were determined to be chemically modified (open purple circles) fall above *C*_max_, while those modulated primarily by electrostatics (open red circles) fall below *C*_max_. We also show *C*_apparent_ for the uncovered gold sample from ref. 12 (open black square), which falls far above *C*_max_.

The maximum mobility that we have achieved in each of our four BN-covered samples is significantly higher than the maximum mobility that we have achieved in any uncovered STO sample (Fig. 3a). The mobility improvement with BN results in part from the added separation between the 2DES and the disordered charges in the electrolyte. As discussed below, we also expect that the BN acts as a barrier to surface-degrading chemical reactions that occur during electrolyte gating or during processing. Our limited sample size produces enough scatter in the maximum mobility as a function of thickness that we cannot identify the main sources of residual disorder.

A single layer of graphene is known to be permeable to protons^30^ but impermeable to other small chemical species, including He atoms^31^ and Li^+^ ions^32^. Because BN has a lattice structure nearly identical to that of graphene, we anticipate a similar diffusion resistance for even our thinnest BN barriers. The energy barrier to diffusion is so high (ref. 32 calculates 10 eV for Li^+^ across graphene) that we still expect impermeability with *V*_gate_ dropped across our BN. An electrolyte-gated gold sample covered by 6 nm of BN behaved in accordance with these expectations: a gold oxide film is readily grown on uncovered gold samples^12^, but the BN-covered gold sample was unmodified (Supplementary Note 3). The chemical species responsible for the redox reaction is unknown, but these results nonetheless illustrate that BN can limit chemical reactions during electrolyte gating.

An intriguing possibility for electrolyte-gated oxides is that BN barriers could prevent oxygen removal. Experiments on rutile TiO_2_ single crystals^10^ and VO_2_ thin films^11^ have found evidence that oxygen near the crystal surface diffuses out through the electrolyte, calling into question the relative roles of oxygen vacancy creation and electrostatic carrier accumulation in tuning the properties of oxide materials. An electrolyte-gating study of STO found that injecting oxygen gas into the electrolyte suppresses the source-drain current, which was interpreted as evidence that the otherwise observed carrier accumulation results from oxygen vacancies^33^. Another study of STO concluded that very high gate voltages are required to create oxygen vacancies, and that the reduced STO system (density ~10^15^ cm^−2^) is three-dimensional and remains conductive at zero gate voltage^14^. While we cannot directly prove the absence of oxygen migration when gating BN-protected STO, we verify that electrostatic carrier accumulation can account for our data by considering the apparent capacitance between the electrolyte and the 2DES, defined as *C*_apparent_=*en*_tot_/*V*_gate_. If electrostatics alone is responsible for the carrier accumulation, a naive model suggests that *C*_apparent_ should fall below a serial arrangement of two capacitances: that of the double layer formed by the ions (12 *μ*F cm^−2^, ref. 34), and that of the BN dielectric. This yields 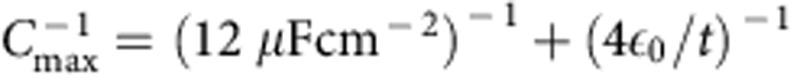, where 4 is the dielectric constant of BN and *t* is the sheet thickness. On the other hand, if carriers accumulate by chemical modification, *C*_apparent_ is unrestricted.

For all BN-covered samples, *C*_apparent_ falls near or below *C*_max_, and two orders of magnitude below *C*_apparent_ for uncovered gold, whose surface is chemically modified by electrolyte gating^12^. The capacitance to the channel from the large coplanar gate, located <200 μm away, accounts for the violation of the electrostatic limit in Samples A (0.6 nm) and C (1.2 nm). Due to the low-temperature dielectric constant of 25,000 in STO and the focusing of field lines from the large gate onto the much smaller Hall bar^35^, this capacitance can be as large as several *μ*Fcm^−2^. We have measured such a capacitance on some samples by zeroing the coplanar gate voltage at low temperature. However, modulating the gate voltage at low temperature appears to cause mechanical problems as our ionic liquid droplet unfreezes on warmup. We therefore did not collect coplanar gate capacitance data for most samples and cannot quantitatively correct *C*_max_.

Our device geometry exposes some area of our contact metal directly to the electrolyte (Fig. 1a), limiting *V*_gate_ to about 3 V: above this, chemical reactions readily occur with the contact metal. This limitation in turn limits the maximum thickness of BN that can be used to create a metallic STO 2DEG. The lowest density for which we have measured a conducting state in our STO 2DES is 10^13^ cm^−2^, although the mobility edge may be somewhat lower. To accumulate 10^13^ cm^−2^ electrostatically requires a minimum capacitance of 0.5 *μ*F cm^−2^, or a maximum BN thickness of 7 nm. Although we have not studied such thick BN flakes, we have measured several samples which had wrinkles in the BN several nm tall due to the transfer process (Supplementary Note 4). When these wrinkles cut fully across the current path between source and drain, the sample never conducted between source and drain. Presumably the area beneath the wrinkles remained insulating, in approximate numerical agreement with the electrostatic accumulation picture.

The BN barrier need not be kept thin if all conductive material can be masked. This is often difficult in insulators, since the electrolyte must create a conductive path between the device channel and metal contacts, unless the insulator can be chemically doped near the contacts. For intrinsically metallic systems, it is straightforward to mask all conductive area (see our BN on gold sample, Supplementary Note 3). In this case, higher voltages can in principle be applied without chemical reactions, increasing the maximum thickness of BN that can be used for a target electron density, which may have advantages for certain materials. Our technique is easily applied to other systems, and should enable electrolyte gating experiments that require high carrier mobility, high carrier density and chemical stability of the surface.

## Methods

### Sample fabrication

Our samples were fabricated on (100) strontium titanate substrates from either Shinkosha Co. (Japan) or Crystec GmbH (Germany); the vendor for each sample is specified in Supplementary Table 1. The surfaces of Shinkosha crystals were TiO_2_-terminated as received. We prepared a nominally TiO_2_-terminated surface on the Crystec samples by the method described in ref. 36. A BN flake was transferred onto the STO surface using the water-based process described in ref. 37, followed by an anneal for 4 h at 500 °C in an Ar/O_2_ atmosphere. An ohmic contact pattern was defined in PMMA via e-beam lithography at 10 kV, after which the sample was milled with Ar ions at 300 V to etch away the exposed BN and about 40 nm of the underlying STO. Ohmic contacts (10 nm titanium, 40 nm gold) were then deposited into the milled trenches by e-beam evaporation. Finally, an insulating mask with holes to expose the Hall bar and the coplanar gate was patterned using 10 kV e-beam lithography. The mask material was either cross-linked PMMA or sputtered alumina; in both cases a negative process was used so that the channel was not exposed to the e-beam.

### Low-temperature measurement

Before measurement of each sample, we cleaned the sample surface of resist residues by a brief exposure to a remote oxygen plasma. We then covered the Hall bar and coplanar gate with a drop of the ionic liquid 1-ethyl-3-methylimidazolium bis(trifluoromethanesulfonyl)amide (EMI-TFSI) and placed the sample inside the vacuum chamber of our cryostat (either a dilution refrigerator with base temperature 40 mK or a variable-temperature insert reaching 350 mK or 1.5 K). We polarized the electrolyte at around 290 K, in either high vacuum or helium vapour, by applying a voltage to the coplanar gate. On cooling, the polarized electrolyte froze and we collected magnetotransport data up to the highest available fields (9, 14, or 31 T) via standard lock-in techniques in a current-biased configuration. We typically used an AC source current of 2 μA, which exceeded the superconducting critical current in all samples, suppressing the superconducting features that would otherwise appear for some cooldowns in Fig. 2 (see also Supplementary Note 5). The sample was then warmed to near room temperature, melting the electrolyte. We always set *V*_gate_ to zero in between cooldowns, which introduces some hysteresis in *V*_gate_.

## Authors contributions

P.G., J.R.W. and D.G.-G. designed the experiment. P.G. fabricated the BN on STO samples and performed the measurements, with help from M.L., S.W.S. and J.R.W. P.G., M.L. and D.G.-G. analysed the data. T.A.P. performed the BN on gold experiment. K.W. and T.T. grew the BN crystals. P.G. prepared the manuscript with input from all authors.

## Additional information

**How to cite this article:** Gallagher, P. *et al*. A high-mobility electronic system at an electrolyte-gated oxide surface. *Nat. Commun*. 6:6437 doi: 10.1038/ncomms7437 (2015).

## Supplementary Material

Supplementary Information Supplementary Figures 1-9, Supplementary Tables 1-2, Supplementary Notes 1-5 and Supplementary References
