# Supplementary material for: A high-mobility electronic system at an electrolyte-gated oxide surface
Source: Nat Commun. 2015 Mar 12;6:6437. doi: 10.1038/ncomms7437 (PMC4382703; doi:10.1038/ncomms7437)
Supplement: Supplementary Information — Supplementary Figures 1-9, Supplementary Tables 1-2, Supplementary Notes 1-5 and Supplementary References [file ncomms7437-s1.pdf]

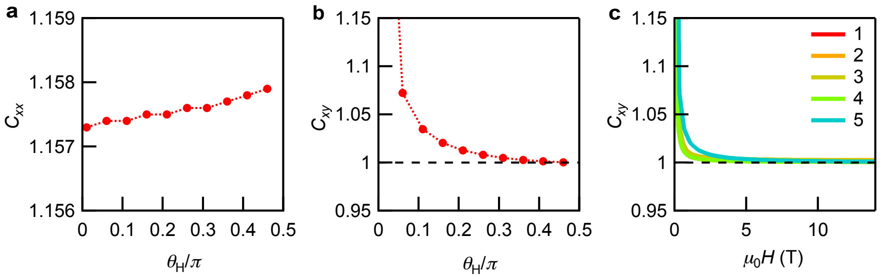

Supplementary Figure 1: Conformal mapping results for Sample A. **a**, Calculated ratio C_{xx} of longitudinal resistance to sheet resistance for Hall angles θ_{H} between 0.01π and 0.46π. **b**, Calculated ratio C_{xy} of transverse resistance to Hall resistance. The black dashed line shows the expected ratio $C_{xy} = 1$ for an ideal Hall bar. Numerical errors in the conformal mapping are most significant near $\theta_{\mathrm{H}} = 0$, and are likely responsible for the deviation of C_{xy} from 1. **c**, The calculation in **b**, interpolated and plotted as a function of applied field for the five cooldowns of Sample A (Figure 2). The cooldown index for each curve is indicated in the legend.

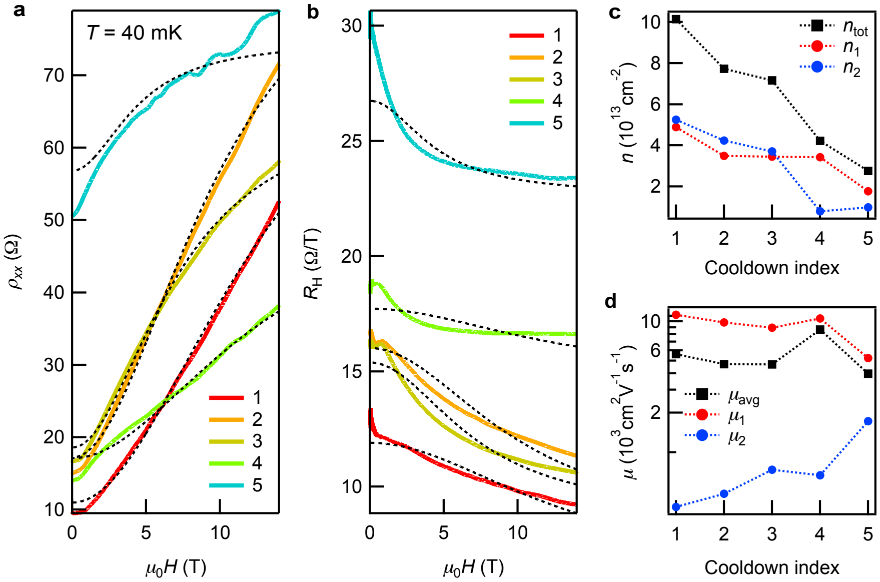

Supplementary Figure 2: Four-parameter, two-band fits to the data in Figure 2. **a**, Sheet resistance ρ_{xx} versus applied magnetic field for the 5 cooldowns in the main text. Black dashed curves are fits to the standard two-band model. **b**, Hall coefficient R_{H} versus applied magnetic field for the same 5 cooldowns. Fits (black dashed curves) are performed simultaneously on the ρ_{xx} and R_{H} data for a given cooldown. **c**, Densities n_1 and n_2 of the two bands; $n_{\mathrm{tot}} = n_1 + n_2$. **d**, Mobilities μ_1 and μ_2; $\mu_{\mathrm{avg}} = (n_1\mu_1 + n_2\mu_2)/n_{\mathrm{tot}}$.

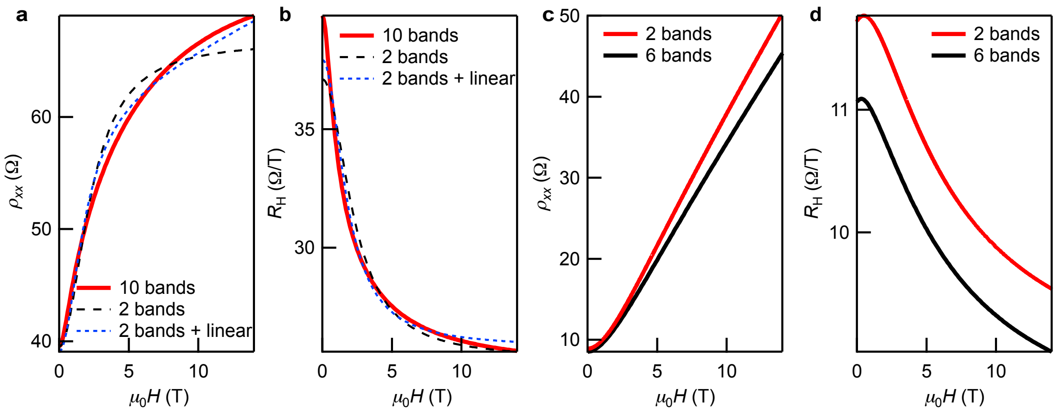

Supplementary Figure 3: Capturing many-band data with a two-band model. **a** and **b**, Simulated sheet resistance ρ_{xx} and Hall coefficient $R_{\rm H}$ versus applied magnetic field for the 10-band system whose parameters are given in Supplementary Table 2 (red curves). Black dashed curves result from a fit to the four-parameter, two-band model. Blue dashed curves result from a fit to the six-parameter, two-band model with linear magnetoresistance. **c** and **d**, Red curves: six-parameter fits to data from Sample A, Cooldown 1. Black curves: same as red curves, except that four low-density bands have been added to the calculation (see text).

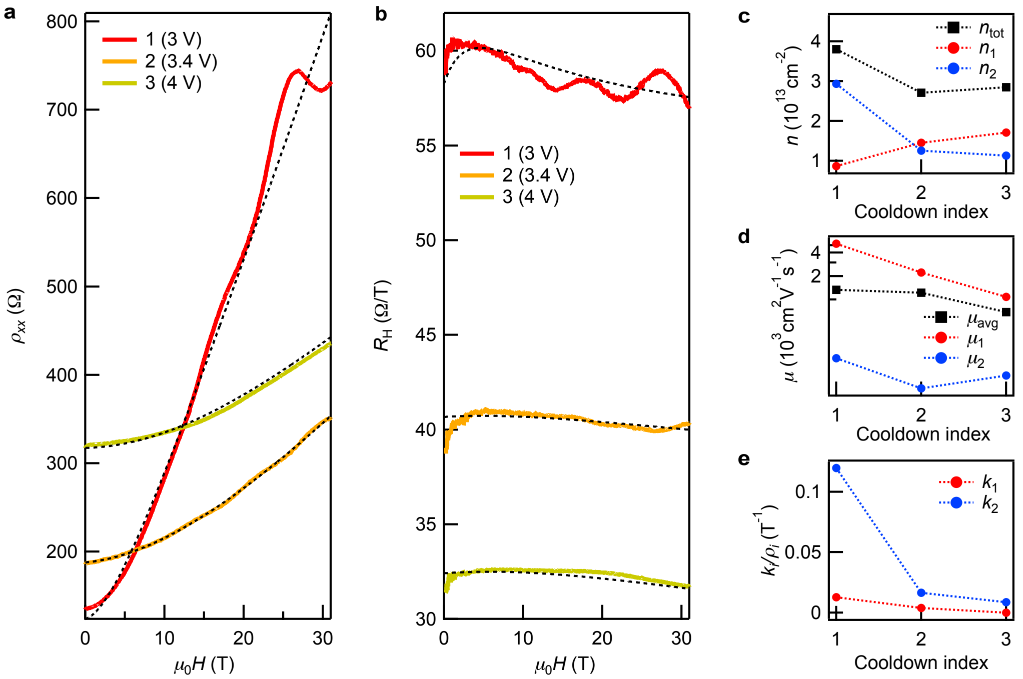

Supplementary Figure 4: Linear magnetoresistance measured up to 31 T in Sample B. **a**, Sheet resistance ρ_{xx} versus applied magnetic field for three successive cooldowns, labeled 1, 2, and 3. The gate voltage used for each cooldown is shown in parentheses in the legend. All cooldowns (but especially Cooldown 1) display nonsaturating and approximately linear magnetoresistance up to the maximum field of 31 T. Black dashed curves are fits to our two-band model with linear magnetoresistance. The sample is immersed in liquid ^{3}He at 350 mK. **b**, Hall coefficient R_H for the three cooldowns. Fits (black dashed curves) are performed simultaneously on the ρ_{xx} and R_H data for a given cooldown. **c**, Densities n_1 and n_2 of the two bands; $n_\mathrm{tot} = n_1 + n_2$. **d**, Mobilities μ_1 and μ_2; $\mu_\mathrm{avg} = (n_1\mu_1 + n_2\mu_2)/n_\mathrm{tot}$. **e**, Linear magnetoresistance coefficients k_i, for $i = 1, 2$, normalized to the zero field resistivities ρ_i of the corresponding bands.

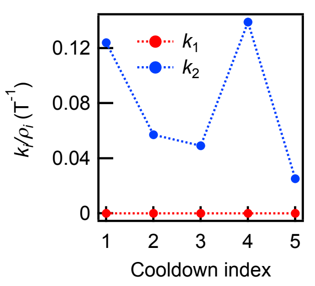

Supplementary Figure 5: Linear magnetoresistance coefficients in Figure 2 fits, normalized to the zero field resistivities ρ_i of the corresponding bands. The fits resulted in a high-mobility band (band 1) with $k_1 = 0$ for all cooldowns.

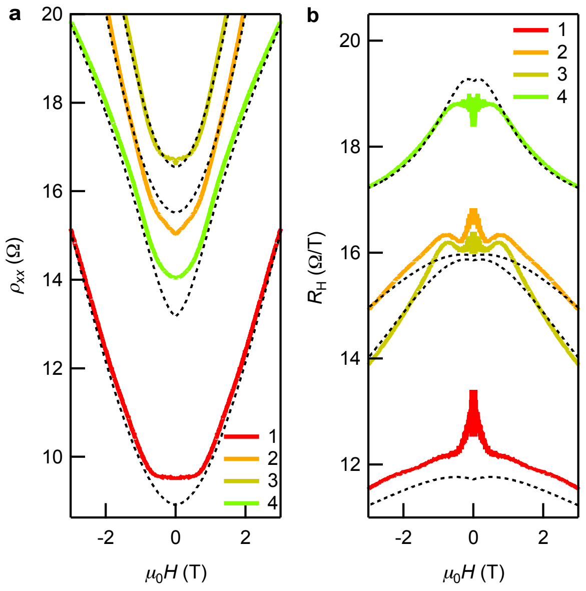

Supplementary Figure 6: Low-field data and fits from Figure 2, for cooldowns 1-4. **a**, Sheet resistance ρ_{xx} and fits (black dashed curves). **b**, Hall coefficient R_{H} and fits (black dashed curves). The ρ_{xx} and R_{H} data cannot be simultaneously fit by our model for fields below ~ 1 T.

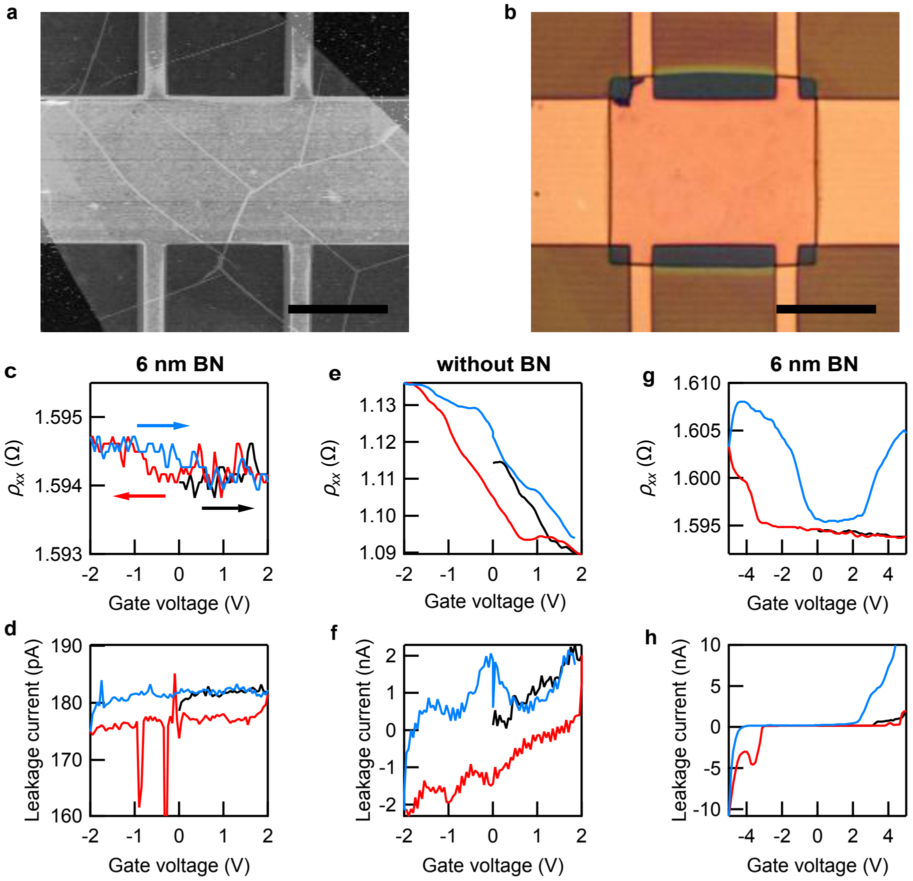

Supplementary Figure 7: Electrolyte gating of gold on SiO_2 with a BN barrier. **a**, AFM topography of the 30-nm-thick gold Hall bar, covered with a 6-nm-thick BN flake, which runs from top left to bottom right. The flake has visible wrinkles and does not conform perfectly to the Hall bar where the channel meets the top two voltage probes. Scale bar: 10 μm. **b**, Optical micrograph of the same device after measurement. A PMMA mask was deposited prior to measurement to ensure that no gold was exposed directly to the electrolyte. The flake is faintly visible beneath the PMMA mask. The upper left voltage lead shows damage (see text). Scale bar: 10 μm. **c**, Sheet resistance ρ_{xx} for a sequence of gate voltage sweeps, in vacuum at 300 K. Black trace: sweeping from 0 to 2 V. Red trace: 2 to -2 V. Blue trace: -2 to 2 V. **d**, Leakage current through the electrolyte for the same sweeps; there is a $\sim$ 180 pA offset in our measurement electronics. The measured leakage current is zero to within our sensitivity. **e** and **f**, Equivalent dataset to **c** and **d** on a gold device of similar thickness but without BN, showing significant leakage current and ρ_{xx} modulation. **e** and **f**, Sheet resistance and leakage current for sweeps between $\pm$5 V, performed after the sweeps in **c** and **d**. The leakage becomes nonzero as the gate reaches 3 V for the first time (black trace); ρ_{xx} begins to change significantly after the gate reaches -3 V.

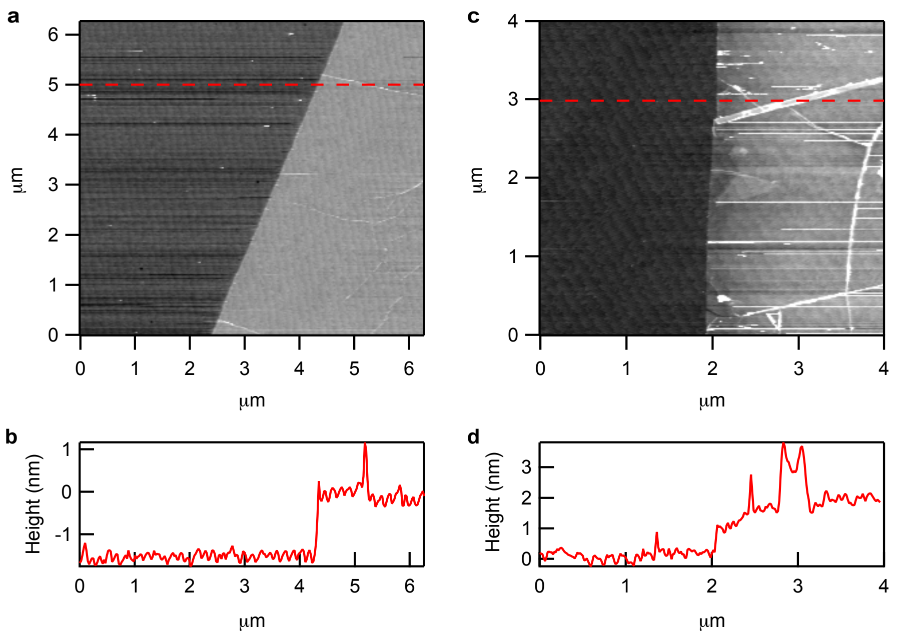

Supplementary Figure 8: Wrinkles in BN flakes transferred to STO. **a**, Contact AFM topography of a flake showing minor wrinkles (right side of image) on STO (left), with typical wrinkle heights 0.5 to 1 nm. **b**, Height data for the scan along the red dashed line in **a**. **c**, Non-contact AFM topography of a much more severely wrinkled flake (right) on STO (left), where wrinkles are as high as 3 nm. The sample produced from this flake did not conduct between contacts, which were separated by thick wrinkles that spanned the channel. **d**, Height data for the scan along the red dashed line in **c**.

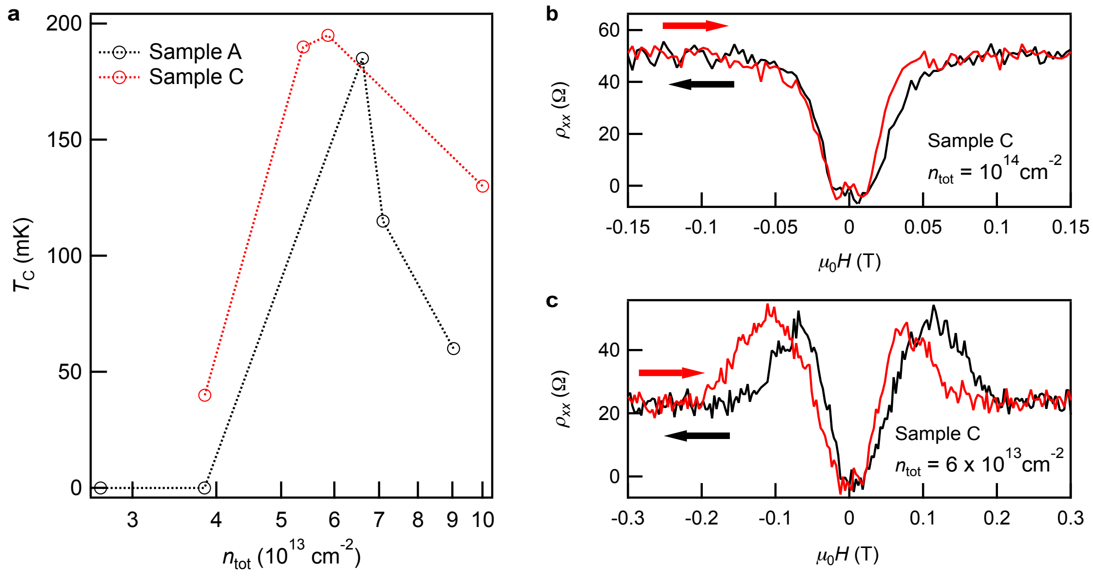

Supplementary Figure 9: Superconducting properties of Samples A and C. **a**, Critical temperature T_C for superconductivity as a function of total carrier density from our fits with linear magnetoresistance. T_C is defined as the temperature at which the resistance drops to half the normal state value. **b**, Sheet resistance of Sample C in a small perpendicular magnetic field for $n_\mathrm{tot} = 10^{14}\ \mathrm{cm}^{-2}$, measured with 5 nA ac excitation current and zero dc current. The black curve was taken while decreasing H, and the red curve was taken while increasing H. Minimal hysteresis is observed, as is typical for most cooldowns. **c**, Sheet resistance of Sample C in a small perpendicular magnetic field for $n_\mathrm{tot} = 6 \times 10^{13}\ \mathrm{cm}^{-2}$, measured with 5 nA ac excitation current and zero dc current. Hysteresis is observed in the resistance peaks near the critical field (see text).

Sample name	Crystal vendor	Mask material	BN thickness	L_{tot}	L_c	W	D_{coplanar}
Sample A	Shinkosha	PMMA	0.6 ± 0.1 nm	23 μm	18.5 μm	14 μm	126 μm
Sample B	Crystec	alumina	1.0 ± 0.5 nm	9 μm	6 μm	7.5 μm	84 μm
Sample C	Shinkosha	PMMA	1.2 ± 0.3 nm	6 μm	4 μm	4 μm	194 μm
Sample D	Crystec	alumina	1.5 ± 0.3 nm	10 μm	7 μm	6 μm	125 μm

Supplementary Table 1: Sample details. L_{tot} is the total length of the Hall bar channel, and W is the channel width. L_c is the center-to-center distance between R_{xx} voltage probes. D_{coplanar} is the distance from the Hall bar to the nearest point on the coplanar gate.

Band index i	n_i (cm^{-2})	μ_i (cm^{2}V^{-1}s^{-1})
1	1×10^{12}	20000
2	2×10^{12}	15000
3	2×10^{12}	12000
4	3×10^{12}	8000
5	3×10^{12}	6000
6	3×10^{12}	5000
7	2×10^{12}	4000
8	3×10^{12}	3000
9	4×10^{12}	2000
10	2×10^{12}	1000

Supplementary Table 2: Parameters for ten bands used to generate data in Supplementary Figure 3. These parameters are chosen to be plausible given the densities extracted from quantum oscillations and the average mobility and total density that we extract from the Hall analysis, but are otherwise arbitrary.

Supplementary Note 1: Sample properties

Lateral dimensions

The samples studied in this work have different lateral dimensions because we are constrained by the dimensions of the chosen BN barrier flake. We are also typically forced to position our coplanar gate and ohmic contacts away from thick, randomly-positioned flakes that are unintentionally transferred alongside the flake of interest, leading to further geometry variations. Nonetheless, the approximate geometry in all cases is as shown in Figure 1a in the main text, with a small Hall bar about 100 μm from a coplanar gate. The gate dimensions are on the order of 1 mm by 0.2 mm: the gate must be much larger than the Hall bar so that the applied voltage drops across the BN, instead of at the double layer at the coplanar gate. Ohmic traces are a few microns wide and extend to bond pads about 1 mm away from the Hall bar and coplanar gate. Some specific sample dimensions are listed in Supplementary Table 1.

Flake thickness

We determined the flake thicknesses reported in Supplementary Table 1 by atomic force microscopy (AFM) after transferring the flakes onto the STO crystal and annealing at 500°C. We found that after annealing,

a low density of small, mobile particles remained on the sample surface. While these particles were too small and too sparse to affect transport measurements, they could easily be collected by the AFM tip and redeposited, particularly at the step edge whose height we wanted to measure. This introduced sizable errors in our tapping mode AFM (TAFM) measurements. The thickness uncertainties quoted in Supplementary Table 1 reflect the measured thickness variations between linescans at different positions on the same flake. Sample B was particularly difficult to image with TAFM because of mobile particles, and therefore has a large error bar.

For our later generation of samples (Samples A and C), we used contact AFM (CAFM), which is more suitable than TAFM for studying a surface with mobile particles, and generally leads to a more accurate step height measurement between substrate and flake.[1,2] However, the difference in friction between STO and few-layer h-BN causes twisting of the CAFM cantilever and thus a different measured flake thickness for left-moving and right-moving scans.[1] Because the offset between left-moving and right-moving scans is small (several Å), to first order the correct step height can be found by averaging the step heights measured in each direction. This average thickness is the thickness quoted in Supplementary Table 1 for Samples A and C, and the quoted uncertainties reflect the variation in average thickness between linescans at different positions on the same flake. As a sanity check, we also performed TAFM on Samples A and C, and found thicknesses consistent with the average thickness measurements from CAFM.

Determination of ρ_{xx} and ρ_{xy}

In this study, the quantities that we directly measure as a function of magnetic field are the longitudinal resistance $R_{xx}(H)$ and the transverse resistance $R_{xy}(H)$ of our six-terminal devices. To extract the sheet resistance $\rho_{xx}(H)$ of the 2DES from the measured signal $R_{xx}(H)$, $R_{xx}(H)$ must be multiplied by a factor that accounts for geometrical details such as the number of squares between the voltage probes, the nonzero width of the voltage probes, and the distance between the voltage probes and the current leads. Similarly, to determine the Hall resistance $\rho_{xy}(H)$, the measured transverse resistance $R_{xy}(H)$ must also be corrected for a geometrical factor, since in some geometries the Hall voltage can be shorted out by the current leads.[3] In general, these geometrical correction factors may depend on the Hall angle $\theta_{\mathrm{H}} = \tan^{-1}(\rho_{xy}(H)/\rho_{xx}(H))$.

We have used the conformal mapping technique[4] to numerically determine the geometrical correction factors $C_{xx} \equiv R_{xx}/\rho_{xx}$ and $C_{xy} \equiv R_{xy}/\rho_{xy}$ for the geometry of each sample. Conformal mapping calculations were performed using the Schwarz-Christoffel Toolbox for MATLAB, created by Tobin Driscoll (`http://www.math.udel.edu/~driscoll/SC/index.html`). For all samples and all experimentally relevant Hall angles θ_{H}, we find that $C_{xy} \approx 1$, and $C_{xx} \approx L_c/W$, where L_c is the center-to-center distance between

longitudinal voltage probes and W is the channel width. We therefore compute $\rho_{xx}(H) = C_{xx}R_{xx}(H)$, where C_{xx} is the field-independent constant calculated by conformal mapping, and we set $\rho_{xy}(H) = R_{xy}(H)$ so that the Hall coefficient $R_{\mathrm{H}}(H) \equiv \rho_{xy}(H)/\mu_0 H = R_{xy}(H)/\mu_0 H$.

For concreteness, we describe in detail the results of the conformal mapping for Sample A. The calculated longitudinal correction factor $C_{xx}(\theta_{\mathrm{H}})$ varies by less than 0.1% over the full range of Hall angles (Supplementary Figure 1a). The calculated $C_{xy}(\theta_{\mathrm{H}})$ converges to 1 as θ_{H} approaches $\pi/2$ (Supplementary Figure 1b); minor deviations from 1 at lower θ_{H} likely result from numerical errors in the conformal mapping, which grow large for smaller θ_{H}. Regardless of the cause of the deviations, most of the experimental field range corresponds to θ_{H} sufficiently large that $C_{xy} = 1$ (Supplementary Figure 1c).

Supplementary Note 2: Fitting details

Fitting procedure

All fitting described in this work has been carried out using the Ceres Solver (`http://ceres-solver.org/`), an open-source C++ library for solving nonlinear least squares problems. Our fitting procedure takes two input datasets, $\rho_{xx}(H)$ and $R_{\mathrm{H}}(H)$, and finds the single set of two-band model parameters that best fits both datasets. We use a least-squares residual in which each term is normalized by the corresponding value of $\rho_{xx}(H)$ or $R_{\mathrm{H}}(H)$, so that the fitting does not favor high-field data over low-field data:

$$\text{Residual} \equiv \sum_{H} \left(\frac{(\rho_{xx}(H) - f_{xx}(H; n_1, n_2, ...))^2}{\rho_{xx}(H)^2} + C \frac{(R_{\mathrm{H}}(H) - f_{\mathrm{H}}(H; n_1, n_2, ...))^2}{R_{\mathrm{H}}(H)^2} \right),$$

where f_{xx} and f_{H} are the two-band predictions for ρ_{xx} and R_{H}, and C is a constant that adjusts the relative importance of R_{H} over ρ_{xx} in the fitting. Typically we set $C = 1$, but for some datasets, in particular those with strong ρ_{xx} oscillations, we increase C to get appropriate fits. The constraints on the fit parameters are very loose: parameters are allowed to range over several orders of magnitude. Our best fits are reproducible over a large range of initial conditions provided to the fitting algorithm.

Linear magnetoresistance model

Our fits presented in the main text use a two-band model in which the resistivity tensor of each band is described by

$$\rho_{xx,i} = \rho_i + k_i \mu_0 H$$
$$\rho_{xy,i} = \mu_0 H / n_i e,$$

where $\rho_i \equiv 1/n_i e \mu_i$ is the zero-field resistivity of band i, n_i is the density of band i, $k_i \geq 0$, and μ_0 is the magnetic constant (in the main text we absorbed μ_0 into k_i for simplicity). The conductivity tensor of the complete system is the sum of the conductivity tensors of the individual bands.

Fitting more than two bands with a two-band model

It is clear from the presence of quantum oscillations with many frequencies that more than two bands contribute to transport. This is also the case for the highest-mobility LAO/STO 2DES, in which several bands contribute to quantum oscillations even for total carrier densities as low as 10^{13} cm^{-2} (Ref. 5, 6). Despite the large number of bands in our 2DES, the two-band model with linear magnetoresistance presented in the text captures the essential magnetotransport behavior. In fact, fits to the data in Figure 2 in the main text are also possible with the four-parameter, two-band model,[7] although the fit quality is lower (Supplementary Figure 2a,b). The extracted densities (Supplementary Figure 2c) and mobilities (Supplementary Figure 2d) are still comparable to those shown in the main text.

The two-band model can give a reasonable fit to the many-band data by averaging the set of high mobility bands into one band, and the set of low-mobility bands into another band. For instance, if we assume the existence of ten bands with the arbitrarily-chosen (but plausible) densities and mobilities shown in Supplementary Table 2, the simulated ρ_{xx} and R_{H} are acceptably fit by the four-parameter, two-band model (Supplementary Figure 3a,b). The resulting fit parameters are $n_1 = 1.1 \times 10^{13}$ cm^{-2}, $n_2 = 1.4 \times 10^{13}$ cm^{-2}, $\mu_1 = 11267$ cm^{2}V^{-1}s^{-1}, and $\mu_2 = 2467$ cm^{2}V^{-1}s^{-1}. These values yield a total density $n_{\mathrm{tot}} \equiv \sum_i n_i = 2.5 \times 10^{13}$ cm^{-2} and an average mobility is $\mu_{\mathrm{avg}} \equiv \sum_i n_i \mu_i / n_{\mathrm{tot}} = 6395$ cm^{2}V^{-1}s^{-1}, which agree well with the total density 2.5 $\times 10^{13}$ cm^{-2} and average mobility 6320 cm^{2}V^{-1}s^{-1} calculated from Supplementary Table 2.

The six-parameter fit with linear magnetoresistance yields better agreement to the ρ_{xx} and R_{H} curves, even though no linear magnetoresistance is used to produce the ten-band curves (Supplementary Figure 3a,b). The fit parameters are $n_1 = 9.2 \times 10^{12}$ cm^{-2}, $n_2 = 1.5 \times 10^{13}$ cm^{-2}, $\mu_1 = 12382$ cm^{2}V^{-1}s^{-1}, $\mu_2 =$

3039 $\mathrm{cm^2V^{-1}s^{-1}}$, $k_1 = 0$, and $k_2 = 1.6\ \Omega/\mathrm{T}$, which give $n_{\mathrm{tot}} = 2.4\times10^{13}\ \mathrm{cm^{-2}}$ and $\mu_{\mathrm{avg}} = 6587\ \mathrm{cm^2V^{-1}s^{-1}}$, also in good agreement with the correct values.

On the other hand, if we assume that there are in fact two higher-density bands in our system, the addition of several low-density oscillating bands does not strongly affect the shapes of the magnetotransport curves. For a specific example, consider adding four bands, each of density $10^{12}\ \mathrm{cm^{-2}}$, with mobilities 5000, 6000, 8000, and 12000 $\mathrm{cm^2V^{-1}s^{-1}}$ to the two bands with linear magnetoresistance extracted in the main text (Figure 2) for Sample A, Cooldown 1. The four additional bands have linear magnetoresistance coefficients of zero, for simplicity. Other than globally shifting R_{H} because of the increased density, the addition of these bands has a modest effect on ρ_{xx} and R_{H} (Supplementary Figure 3c,d).

We conclude that regardless of the actual number of bands, the two-band model with or without linear magnetoresistance is a reliable method for extracting total density and average mobility.

Justification of two-band fits with linear magnetoresistance

The fact that the six-parameter fit could more closely approximate the ten-band ρ_{xx} and R_{H} (Supplementary Figure 3a,b) might suggest that the linear terms that we have introduced in these fits are not physically meaningful. However, in many of our samples we observe a nonsaturating, approximately linear ρ_{xx} despite nearly saturating R_{H}. Such behavior is particularly evident for Sample A, Cooldown 4; here the data are poorly fit by the four-parameter, two-band model (Supplementary Figure 2), but very well fit by the six-parameter, two-band model (Figure 2).

The linear term is further justified by our observation in Sample B of nonsaturating linear magnetoresistance despite nearly saturated R_{H} up to fields as high as 31 T (Supplementary Figure 4a) at the National High Magnetic Field Lab. Sample B is the only sample that we measured in the Magnet Lab. Although this sample exhibits lower mobility (Supplementary Figure 4d) than other BN-covered STO samples, which we ascribe to subpar cleanliness of our procedures away from our home lab, the qualitative magnetoresistance behavior is similar to that of our other samples measured at lower fields. Nonsaturating linear magnetoresistance with an apparently saturating R_{H} has also been observed in LAO/STO up to 31 T (Ref. 8).

We have attempted to fit our data from all samples with a three-band model (parameters n_1, n_2, n_3, μ_1, μ_2, μ_3), since a high-density, low-mobility band could in principle give rise to a nearly linear growth of ρ_{xx} with a nearly constant R_{H} for the experimentally-accessible field range. However, the three-band model does not fit all of our datasets, while the two-band model with linear magnetoresistance can generally fit the data very well. Even when the three-band fits do capture the data, the resulting densities are surprisingly

high, ranging from 10^{14} to 10^{16} cm^{-2}, with mobilities ~ 1 cm^{2}V^{-1}s^{-1}. The two-band model with linear magnetoresistance is our best known method to accurately fit our data.

Interpretation of k_i

In our six-parameter, two-band fits with linear magnetoresistance coefficients k_i, we typically find that the high-mobility band has a smaller linear coefficient than does the low-mobility band, even when k_i is normalized to the zero-field resistivity ρ_i (Supplementary Figure 4e). In fact, our fits in Figure 2 in the main text all yielded exactly zero for k_1, the linear magnetoresistance coefficient of the high-mobility band (Supplementary Figure 5). One possible interpretation of this result, assuming that the linear magnetoresistance arises from spatial fluctuations in the mobility,[9,10] is that the low-mobility band is nearer the STO surface, while the high-mobility band is buried deeper. The inhomogeneities responsible for the mobility fluctuations plausibly reside on the surface, thereby more strongly affecting the low-mobility band.

However, we caution that the linear term is only the first-order correction to the band resistivities $\rho_{xx,i}$. The linear magnetoresistance due to mobility fluctuations exhibits a crossover field, below which the magnetoresistance is quadratic.[9,10] Giving the linear magnetoresistance mainly to the low-mobility band may simply help approximate this shape: if the densities of the two bands are similar, the low-mobility band becomes relevant to the magnetoresistance only at higher fields.

Low-field data

All of the fits shown in the main text and in the Supplementary Information neglect the low-field region, where magnetic effects are likely important.[11] We find that the behavior of ρ_{xx} and R_{H} in this region cannot be simultaneously captured by few-band physics, even with linear magnetoresistance terms (Supplementary Figure 6). Cooldowns 1, 2, and 3 had superconducting features, with $T_{\mathrm{C}} = 60$, 115, and 185 mK, respectively. These features do not appear in Supplementary Figure 6 because the critical current in each case is much smaller than the 2 μA ac excitation. When measured with a smaller ac excitation, the superconducting features are suppressed above $H_{\mathrm{C}} \sim 50$ mT.

Supplementary Note 3: Chemical resistance of BN flakes

An electrolyte-gating study of gold films has reported carrier density modulation of up to 3×10^{15} cm^{-2} and correspondingly large changes in sheet resistance.[12] The capacitance required for these changes exceeds by an order of magnitude the expected capacitance of the electrolyte gate,[13] suggesting that chemical modification

of the gold film is responsible for the modulation. Indeed, we have shown that electrolyte gating readily oxidizes the gold film at negative gate voltages, and that this oxide growth accounts for at least 90% of the observed changes in transport properties.[14]

To determine whether our BN films can prevent this sort of electrochemical reaction, we transferred a BN flake 6 nm thick onto a gold Hall bar 30 nm thick. The topography scan of the resulting structure shows that the flake mostly conforms to the Hall bar (Supplementary Figure 7a). However, because the Hall bar protrudes 30 nm from the oxide substrate, the flake relaxes strain by forming several wrinkles. The flake is also not perfectly flush with the gold where top two voltage probes meet the channel. Prior to electrolyte gating, all unprotected gold areas were masked by a layer of crosslinked PMMA (Supplementary Figure 7b). The protected Hall bar and the on-chip coplanar gate were then covered in the ionic liquid N,N-diethyl-N-methyl-N-2-methoxyethyl ammonium tetrafluoroborate (DEME-BF4) and inserted in a vacuum chamber at room temperature.

First, we swept the gate voltage between -2 and 2 V. The total change in ρ_{xx} over these 4 V is $\sim 5\times10^{-4}\ \Omega$ (Supplementary Figure 7c). For 4 V dropped across a BN flake 6 nm thick, electrostatics gives a density change $\Delta n = 4\epsilon_0 \times 4$ V/6 nm $= 1.5 \times 10^{13}$ cm^{-2} (we neglect the double layer capacitance, which is at least ten times the capacitance from the 6 nm BN dielectric). The total 2D carrier density in a gold film 30 nm thick is $n = 1.8 \times 10^{17}$ cm^{-2}. If the sheet resistance is proportional to $1/n$, electrostatics predicts a fractional change in sheet resistance $\sim 10^{-4}$, close to the fractional change $\sim 3 \times 10^{-4}$ that we observe. Considering that we have neglected changes in electron mobility and the inhomogeneous distribution of the induced carrier density within the gold film, this approximate agreement is consistent with purely electrostatic carrier accumulation. The gate leakage current through the electrolyte and into the sample (Supplementary Figure 7d) is well below the 10 pA resolution of our measurement electronics, further implying the absence of electrochemical reactions, which would involve charge transfer from the electrolyte to the sample.

In contrast, electrolyte gating of an unprotected gold Hall bar with similar film thickness shows a very strong modulation of ρ_{xx} and a large leakage current even for very small gate voltages (Supplementary Figure 7e,f). The fractional change in ρ_{xx} over the -2 to 2 V window is ~ 130 times larger for the uncovered sample than it is for the covered sample. This cannot be fully accounted for by the added thickness of the BN spacer, which should decrease the parallel plate capacitance from the double layer value of 7 μFcm^{-2} (Ref. 14) by a factor of 12. Thus, the fractional change in ρ_{xx} for a given electric field is 10 times smaller in the BN-protected sample than it is in the unprotected sample, implying that the BN very strongly suppresses, if not entirely prevents, the oxidation of gold by the electrolyte.

We next proceeded to higher gate voltages on our BN-covered sample. In our first sweep up from 0 to 5 V, the leakage current started to noticeably increase at around 3 V (Supplementary Figure 7h), but the

change in ρ_{xx} remains small (Supplementary Figure 7g). However, upon sweeping from 5 to -5 V, we find a sudden ρ_{xx} increase around -3 V, coincident with a leakage current spike. The next sweep, from -5 to 5 V, shows a significant ρ_{xx} modulation even for positive gate voltages. The fractional magnitude of this modulation per volt is comparable to that of the unprotected gold Hall bar (Supplementary Figure 7e).

These data strongly suggest that our BN flake effectively blocks the oxidation reaction at the gold surface. At a sufficiently high voltage (approximately $\pm$ 3 V), the BN barrier itself starts to degrade, allowing reactions with the gold surface. The damage to the BN flake and/or the underlying gold is visible in an optical micrograph of the device after measurement (Supplementary Figure 7b), which shows shows dark spots in the upper left corner of the channel where prior to measurement the BN flake was wrinkled and poorly conformed to the gold surface. We expect that BN flakes without such mechanical defects will withstand a larger voltage before degrading.

Supplementary Note 4: Wrinkles in BN flakes

The BN flakes in this study are transferred using a PMMA membrane.[15] This process leaves a layer of polymer residue all over the sample, even after cleaning in standard solvents. We remove this residue by annealing in oxygen at 500°C. One consequence of this is that the flakes tend to wrinkle, perhaps because of the different thermal expansion coefficients of STO and BN, or because small pockets of trapped contaminants escape from beneath the BN. If these wrinkles are only $\sim$ 1 nm tall (Supplementary Figure 8a,b), they present no obvious problem to our measurements: our highest mobility sample (Sample A) has small wrinkles, while Sample C has no wrinkles in the channel but still a lower mobility. However, some flakes have wrinkles several nm high (Supplementary Figure 8c,d); wrinkles this tall appear to prevent the underlying STO from being metallic at low temperature, just as one would expect based on electrostatic carrier accumulation.

The flakes as exfoliated are typically not wrinkled. The wrinkling is a byproduct of our particular transfer method and annealing process. Other methods, such as the dry transfer process based on polypropylene carbonate (PPC), are capable of wrinkle-free transfers.[16] We have found in our limited experience with the PPC-based process that PPC leaves less residue than does PMMA. More importantly, this PPC residue can be removed with a remote oxygen plasma, avoiding the need for annealing.

Supplementary Note 5: Superconducting properties of BN-protected STO samples

Samples A and C were measured under appropriate density and temperature conditions to observe superconductivity. Although the critical temperature for superconductivity T_C in our samples does appear to be maximized near a total density $n_{tot} = 6 \times 10^{13}$ cm^{-2} (Supplementary Figure 9a), we emphasize that other factors such as carrier mobility[17] may strongly affect to T_C. The magnitude of the critical current varies with sample size and T_C. For Sample C, which is 4 microns wide, at optimal doping (T_C = 195 mK) the critical current is 200 nA.

When sweeping a perpendicular magnetic field, we typically observe minimal hysteresis (Supplementary Figure 9b). On some cooldowns of Sample C, we find a resistance peak near the critical field (Supplementary Figure 9c) and critical temperature at zero dc current. Similar behavior has been observed in mesoscopic aluminum samples, and has been ascribed to various mechanisms such as an inhomogeneous distribution of normal metal-superconductor interfaces[18] or vortex dynamics that depend on the sample geometry.[19] While these resistance peaks in our sample show significant hysteresis (Supplementary Figure 9c), we hesitate to ascribe this hysteresis to magnetism or other physical effects in the STO: the hysteresis could simply reflect demagnetization cooling in our probe, which we have not carefully tested.

Supplementary References

[1] Nemes-Incze, P., Osvth, Z., Kamars, K. & Bir, L. Anomalies in thickness measurements of graphene and few layer graphite crystals by tapping mode atomic force microscopy. *Carbon* **46**, 1435–1442 (2008).

[2] Lee, C. *et al.* Frictional characteristics of atomically thin sheets. *Science* **328**, 76–80 (2010).

[3] Look, D. C. *Electrical Characterization of GaAs Materials and Devices* (Wiley, New York, 1989).

[4] Wick, R. F. Solution of the Field Problem of the Germanium Gyrator. *J. Appl. Phys.* **25**, 741–756 (1954).

[5] McCollam, A. *et al.* Quantum oscillations and subband properties of the two-dimensional electron gas at the $LaAlO_3/SrTiO_3$ interface. *APL Mater.* **2**, 022102 (2014).

[6] Xie, Y. *et al.* Quantum longitudinal and Hall transport at the $LaAlO_3/SrTiO_3$ interface at low electron densities. *Solid State Commun.* **197**, 25–29 (2014).

[7] Joshua, A., Pecker, S., Ruhman, J., Altman, E. & Ilani, S. A universal critical density underlying the physics of electrons at the $LaAlO_3/SrTiO_3$ interface. *Nat. Commun.* **3**, 1129 (2012).

[8] Ben Shalom, M., Ron, A., Palevski, A. & Dagan, Y. Shubnikov-De Haas Oscillations in $SrTiO_3/LaAlO_3$ Interface. *Phys. Rev. Lett.* **105**, 206401 (2010).

[9] Parish, M. & Littlewood, P. Non-saturating magnetoresistance in heavily disordered semiconductors. *Nature* **426**, 162–165 (2003).

[10] Kozlova, N. V. *et al.* Linear magnetoresistance due to multiple-electron scattering by low-mobility islands in an inhomogeneous conductor. *Nat. Commun.* **3**, 1097 (2012).

[11] Joshua, A., Ruhman, J., Pecker, S., Altman, E. & Ilani, S. Gate-tunable polarized phase of two-dimensional electrons at the $LaAlO_3/SrTiO_3$ interface. *PNAS* **110**, 9633–9638 (2013).

[12] Daghero, D. *et al.* Large Conductance Modulation of Gold Thin Films by Huge Charge Injection via Electrochemical Gating. *Phys. Rev. Lett.* **108**, 066807 (2012).

[13] Bard, A. J. & Faulkner, L. R. *Electrochemical Methods: Fundamentals and Applications*, 2nd edn (Wiley, New York, 2001).

[14] Petach, T. A., Lee, M., Davis, R. C., Mehta, A. & Goldhaber-Gordon, D. Mechanism for the large conductance modulation in electrolyte-gated thin gold films. *Phys. Rev. B* **90**, 081108 (2014).

[15] Amet, F., Williams, J. R., Watanabe, K., Taniguchi, T. & Goldhaber-Gordon, D. Insulating Behavior at the Neutrality Point in Single-Layer Graphene. *Phys. Rev. Lett.* **110**, 216601 (2013).

[16] Wang, L. *et al.* One-dimensional electrical contact to a two-dimensional material. *Science* **342**, 614–617 (2013).

[17] Bell, C. *et al.* Dominant Mobility Modulation by the Electric Field Effect at the $LaAlO_3/SrTiO_3$ Interface. *Phys. Rev. Lett.* **103**, 226802 (2009).

[18] Arutyunov, K., Presnov, D., Lotkhov, S., Pavolotski, A. & Rinderer, L. Resistive-state anomaly in superconducting nanostructures. *Phys. Rev. B* **59**, 6487–6498 (1999).

[19] Harada, A. *et al.* Sample size dependence of excess resistance near critical field in mesoscopic superconducting Al disk. *J. Phys.: Conf. Ser.* **150**, 022022 (2009).
